# Lassa virus glycoprotein complex review: insights into its unique fusion machinery

**DOI:** 10.1042/BSR20211930

**Published:** 2022-02-14

**Authors:** Hallie N. Pennington, Jinwoo Lee

**Affiliations:** Department of Chemistry and Biochemistry, College of Computer, Mathematics, and Natural Science, University of Maryland College Park, College Park, MD 20740, U.S.A.

**Keywords:** arenavirus, glycoproteins complex, Lassa Virus, membrane fusion

## Abstract

Lassa virus (LASV), an arenavirus endemic to West Africa, causes Lassa fever—a lethal hemorrhagic fever. Entry of LASV into the host cell is mediated by the glycoprotein complex (GPC), which is the only protein located on the viral surface and comprises three subunits: glycoprotein 1 (GP1), glycoprotein 2 (GP2), and a stable signal peptide (SSP). The LASV GPC is a class one viral fusion protein, akin to those found in viruses such as human immunodeficiency virus (HIV), influenza, Ebola virus (EBOV), and severe acute respiratory syndrome coronavirus 2 (SARS-CoV-2). These viruses are enveloped and utilize membrane fusion to deliver their genetic material to the host cell. Like other class one fusion proteins, LASV-mediated membrane fusion occurs through an orchestrated sequence of conformational changes in its GPC. The receptor-binding subunit, GP1, first engages with a host cell receptor then undergoes a unique receptor switch upon delivery to the late endosome. The acidic pH and change in receptor result in the dissociation of GP1, exposing the fusion subunit, GP2, such that fusion can occur. These events ultimately lead to the formation of a fusion pore so that the LASV genetic material is released into the host cell. Interestingly, the mature GPC retains its SSP as a third subunit—a feature that is unique to arenaviruses. Additionally, the fusion domain contains two separate fusion peptides, instead of a standard singular fusion peptide. Here, we give a comprehensive review of the LASV GPC components and their unusual features.

## Introduction

The *Mammarenavirus* genus of the *Arenaviridae* family comprises a diverse group of enveloped, negative-sense single-stranded RNA viruses and can be divided into two categories, Old-World (OW) or New-World (NW), based on phylogenetic differences and geographical distribution [1,2]. OW arenaviruses are those that circulate in Africa, Asia and Europe, whereas those classified as NW circulate in the Americas [3]. Various species of rodents are the natural reservoirs for these viruses with endemicity dependent on the species’ indigenous location. Several viruses in this family are pathogenic in humans and are causative agents of viral hemorrhagic fevers [4]—one of which, Lassa virus (LASV), an OW arenavirus, will be the focus of this review article.

LASV is endemic to West Africa and is the most prevalent arenavirus afflicting humans [5,6]. It is primarily spread by direct contact with excrement from infected *Mastomys natalensis* rodents and, rarely, between humans by bodily fluids [7–12]. Infection with LASV causes Lassa fever, a hemorrhagic fever with high morbidity and mortality that affects an estimated 100000–300000 individuals annually [10–15]. The case fatality rate (CFR) of LASV historically averages ∼20% [16–19]. For perspective, the severe acute respiratory syndrome coronavirus 2 (SARS-CoV-2) pandemic CFR averaged between 1 and 3% [20–25]. Despite the high pathogenicity of LASV, there are currently no FDA-approved vaccines or antivirals for the explicit treatment of Lassa fever [13,26–29]. Therapeutic options are limited to off-label usage of the antiviral ribavirin, which is only effective in the early stages of infection and has severe side effects, and induction of passive immunity with survivors’ antibodies [11–13,29–32]. Several small-molecule compounds that inhibit arenavirus entry have recently been described and show promise *in vitro* but have not yet advanced past laboratory studies [28,33–42]. Recently, the attenuated Candid#1 vaccine was shown to be effective against Junín virus (JUNV), an NW arenavirus that is the causative agent of Argentine hemorrhagic fever [43,44]. However, the vaccine is ineffective against LASV [45–49]. This has largely been attributed to ample glycosylation of the LASV glycoprotein complex (GPC) that provides immunological resistance [50–54]. Given the relatively high CFR of LASV infections combined with its high infectivity and minimal treatment options, this virus has the potential to be a great risk to public health following the completion of the zoonotic jump. Accordingly, LASV has been categorized as a category A, biosafety level 4 agent by the Centers for Disease Control and Prevention [55]. The World Health Organization also lists LASV as one of the top five infectious diseases requiring prioritized research due to its threat to public health [56,57].

**Figure 1:**
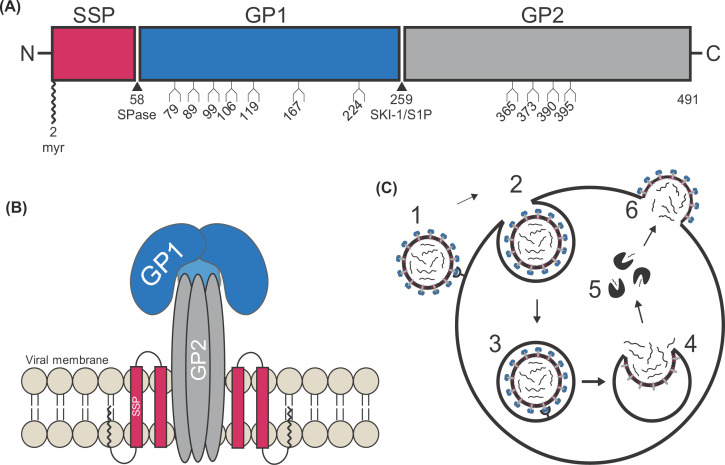
Schematic representation of the LASVGPC (**A**) The GPC open-reading frame is diagrammed with the cleavage sites of SPase and SKI-1/S1P indicated. In SSP (pink), the myristoylation at glycine 2 is marked. The eleven N-glycosylation motifs are denoted at their respective locations in the mature GP1 (dark blue) and GP2 (gray) subunits. (**B**) Cartoon illustration of the GPC trimer as it would appear embedded with in the viral membrane (tan). (**C**) An illustration of GPC’s involvement in the LASV viral lifecycle. GP1 binds to the host receptor (1) before being endocytosed (2). Upon internalization, the virion is delivered to the late endosome where GP1 engages with the intracellular receptor LAMP1 (3). GP1 dissociates so that GP2 is exposed and can mediate membrane fusion leading to the delivery of the viral genome into the host cell cytoplasm (4). The genome is directed to the endoplasmic reticulum where it is transcribed, translated, and replicated (5) before being trafficked to the plasma membrane for assembly and budding (6). Abbreviations: LAMP1, lysosomal-associated membrane protein 1; SPase, cellular signal peptidase.

Delivery of LASV’s genetic material into the host cell is an integral component of its lifecycle. LASV contains a class one fusion protein, akin to those found in human immunodeficiency virus (HIV), Ebola virus (EBOV), SARS-CoV-2, and influenza. These viruses are enveloped and must utilize membrane fusion to deliver their genetic material. The generally accepted model for this process involves the formation of a fusion pore between the host and viral membranes [58–65]. This process is achieved by a glycoprotein located on the virion surface that undergoes a dramatic, energetically favorable conformational change [66–70]. LASV fusion is mediated by the surface GPC, which exists on the virion surface as a trimer of tripartite GPC monomers [71]. GPC is initially expressed as a single polypeptide and undergoes proteolytic cleavage to yield three subunits: glycoprotein 1 (GP1), glycoprotein 2 (GP2), and the stable signal peptide (SSP) (Figure 1A,B) [62,72]. GP1 interacts with host cell receptors, GP2 facilitates membrane fusion, and SSP has various roles, including sensing pH changes to prompt fusion under appropriate conditions, stabilizing GP2’s intermediate conformation, and intracellular trafficking of the GPC precursor [73–75].

**Figure 2:**
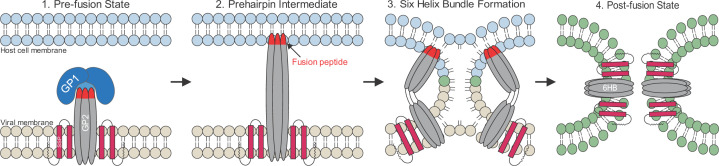
Membrane fusion illustration (1.) The pre-fusion structure of GPC in the viral membrane (tan) is shown. (2.) Upon delivery to the acidic lysosome, GP1 (dark blue) engages with LAMP1 resulting in its dissociation from GPC as triggered by the low pH. GP2 (light gray) becomes unclamped such that the fusion peptide and internal fusion loop at the N-terminus of GP2 (red) are now exposed and inserted into the lysosomal membrane (light blue), forming a metastable pre-fusion hairpin. (3.) GP2 then folds back on itself in an energetically favorable rearrangement. This forms the 6HB and brings the host cell and viral membranes in proximity. (4.) The two membranes then fuse (green), which forms a complete fusion pore and final post-fusion state that exists as a trimer of GP2-SSP. From here the viral genome is released into the cytosol.

LASV follows the general *Arenaviridae* lifecycle (Figure 1C) where host cell entry is mediated by engagement with a host cell receptor, followed by endocytosis [76–80]. During viral entry, GP1 is responsible for binding at least two different cellular receptors: α-dystroglycan (αDG) followed by lysosomal-associated membrane protein 1 (LAMP1). This receptor switch is pH-dependent and unique to LASV as it is not observed in any other arenaviruses [79]. GP1 first engages with the host cell receptor αDG that is located on the plasma membrane. The virion is then internalized and delivered to the late endosome, which is where the membrane fusion event ultimately transpires. The low-pH environment of this compartment triggers GP1 to undergo conformational changes that cause it to now engage with the intracellular receptor LAMP1. This leads to the dissociation of GP1 from the GPC, which results in the exposure of the fusion peptide at the N-terminus of GP2 [77,80–82]. The fusion peptide inserts into the lysosomal membrane, resulting in a metastable pre-hairpin structure, which spontaneously folds back on itself to form a six-helix bundle (6HB), overcoming the energy barrier associated with the fusion of the host and viral membranes. The formation of the 6HB brings the virion and lysosomal membranes in proximity leading to the opening of a fusion pore and delivery of the viral genome into the host cell cytoplasm (Figure 2) [60,82].

GPC and its components prove an interesting target for potential therapeutics owing to its location on the surface of the viral envelope and its central role in membrane fusion, a required step in the viral life cycle. Interestingly, compared with other class one fusion proteins, LASV has several unique structural features including the receptor switch from αDG to LAMP1 as mediated by GP1 and the resulting low pH for optimal fusion. Moreover, another unique feature of LASV is that the fusion peptide contains two distinct components: an N-terminal fusion peptide (NFP), similar to HIV and influenza, and an internal fusion loop (IFL) with a disulfide bond, similar to EBOV. The only other class one fusion protein shown to have both an NFP and IFL are coronaviruses [83–88]. The unusual retention of SSP also has intriguing roles in GPC synthesis and maturation as well as fusion activity [74,75,89–91]. Due to the existence of several excellent review articles on LASV epidemiology, life cycle, and genomic organization [92–98], we will be focusing our discussion on the GPC components and their distinctive properties as related to membrane fusion. All arenaviruses have strong sequence conservation and use a similar mechanism to enter the host cell. As a result, viruses within the same category are often cross-analyzed. Therefore, this review will use evidence from LASV and other arenaviruses, such as lymphocytic choriomeningitis virus (LCMV), another OW arenavirus that is genetically and serologically similar to LASV, and JUNV. For clarity purposes, any non-LASV residues will be followed by the corresponding LASV residue in parentheses.

## Discussion

### Maturation of the GPC yields three components

To fully comprehend the uniqueness of the LASV GPC components, one must first understand the intricacies leading to their development. Like other class one glycoproteins, GPC is first synthesized as a precursor polypeptide that is subsequently cleaved by host cell peptidases to yield the mature GPC. For LASV, GPC is synthesized from the RNA genome as a precursor that is directed to the endoplasmic reticulum. Here, the mature SSP is created via cellular signal peptidase (SPase) cleavage but remains noncovalently associated with the GPC [89]. At the same time, the GPC precursor is translated and undergoes N-linked glycosylation. There are 11 N-glycosylation motifs (N–X–S/T where X is any amino acid, except proline) within the LASV GPC with seven located in GP1 and four in GP2 (Figure 1A) [71,99]. The four glycosylation sites within GP2 are highly conserved across most arenaviruses, whereas the number of glycosylation sites in GP1 varies considerably. This extensive amount of N-linked glycosylation is often referred to as a glycan shield and leaves few regions open to antibody binding. Glycosylation therefore has implications in the arenavirus’ ability to evade antibody-mediated immune responses as the epitope is masked [50–54,62]. These glycosylation motifs are also involved in GP1 receptor recognition, which will be examined in detail shortly [100–102].

For all class one glycoproteins, the precursor fusion protein must be primed via proteolytic cleavage before it can be triggered to induce fusion. For LASV, this priming event occurs after glycosylation and SSP cleavage when the GPC is translocated to the Golgi apparatus as directed by the SSP. Here, the precursor GPC undergoes post-translational proteolytic cleavage by subtilase/kexin isozyme-1 (SKI-1/S1P) to yield the mature GPC containing the GP1 and GP2 components [75,103–106]. For other class one glycoproteins, the signal peptide’s function has been served following final protein localization, so it is degraded and not retained in the mature glycoprotein [60]. Interestingly, the LASV SSP remains associated with the mature GPC—a feature that is unique to arenaviruses and will thus be covered in depth later in this review article. The SSP, GP1, and GP2 are noncovalently bound to each other and form a novel structure not observed in other class one fusion proteins (Figure 1B) [107]. The GPC is then trafficked to the cellular plasma membrane for virion assembly and budding, where it is incorporated on to the surface of the viral envelope (Figure 1C) [108,109]. It is this mature GPC and its components that are responsible for LASV entry.

### Receptor binding occurs via GP1

**Figure 3:**
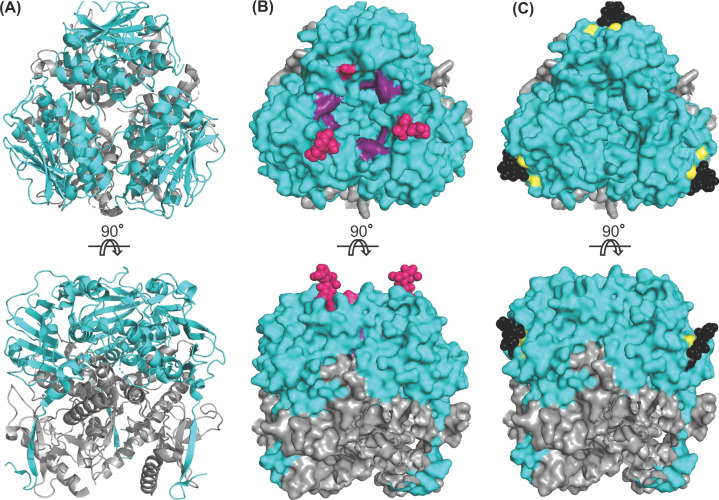
Crystal structure (PDB: 5VK2) of LASV GP1 trimer from the top (upper) and side (lower) views (**A**) Cartoon representation of GP1 (cyan) as it appears when associated with GP2 (gray). (**B**) A surface model of GP1 is presented with the RBD1 highlighted. Residues implicated in αDG-binding cluster in the top central core of GP1, forming the αDG-binding site (purple). The glycosylation motif at N119 (pink) is located near this site but does not occlude it. It is important to note that the crystal structure lacks a glycan in the third GP1 subunit, which would be present* in vivo*. (**C**) The RBD2 of GP1 is represented via a surface model. A histidine triad (yellow) forms the LAMP1-binding site. Protonation of this triad leads to a conformational change that exposes the RBD2. However, this triad is shielded by the glycosylation motif at N89 (black) in the pre-fusion state, which particularly packs against H92. At low pH, this glycan is shifted away to allow for LAMP1 binding. Abbreviation: RBD1/2, receptor-binding domain 1/2.

LASV entry is a multistep process that is first facilitated by GP1, which is 200 amino acids in length and spans from T^59^ to L^259^ of the mature GPC. Initially, GP1 binds with a host cell receptor located on the plasma membrane, specifically the α subunit of dystroglycan (Figure 3A) [110,111]. After binding to αDG, LASV is endocytosed into the host cell, then delivered to the late endosome where it engages with the intracellular receptor LAMP1. This receptor switch is unique to LASV and not observed in other arenaviruses.

#### LASV first engages with host cell receptors at the plasma membrane to enter the host cell

Dystroglycan is a highly conserved cell-surface glycoprotein that is synthesized as a single protein before undergoing post-translational cleavage to yield αDG, the peripheral protein, and βDG, the transmembrane protein [112–115]. It is involved in extracellular matrix adhesion and is a critical component for membrane integrity in numerous cell types, including nerve and muscle cells. In the host cell, αDG is subjected to extensive post-translational modifications, including glycosylation that is critical for its function as an extracellular matrix receptor and arenavirus-binding site [111,116–124]. Regardless, the expression of functional αDG has been demonstrated to be insufficient for productive entry of LASV, but the exact factors that influence entry remain unclear [125]. In the absence of αDG, it has been suggested that GP1 can interact with heparan sulfate, dendritic cell-specific intercellular adhesion molecule-3-grabbing nonintegrin (DC-SIGN), liver and lymph node sinusoidal endothelial cell C-type lectin (LSECtin), and Tyro3/Axl/Mer (TAM) family members to gain entry—albeit with reduced infectivity [78,126–130]. Interestingly, these receptors have been shown to play a role in the entry of several other viruses, including EBOV, dengue virus, ZIKA virus, and SARS coronaviruses [131–138]. This highlights the remarkable adaptability of LASV in the entry process.

Mutational analysis of GP1 has highlighted a potential receptor-binding domain (RBD1) at the top central core of the GPC trimer that is important in αDG binding [139]. Residues H141, N146, F147, and Y150 cluster in this core interface and were implicated in the αDG-binding site (Figure 3B, purple) [139]. Coincidentally, the glycosylation motif at N119 is located near this binding site but does not occlude it (Figure 3B, pink) [50,51]. An N119Q mutation revealed that this glycan protects the αDG-binding site from antibody-mediated neutralization by shielding the epitope [50,53]. The necessity of glycosylation for efficient αDG binding by GP1 has also been validated by inhibition experiments [140]. Additionally, residues located in the same region at the top central core of the LCMV GP1 were implicated in αDG binding [141–143]. This includes S153 (N148), Y155 (Y150), R190 (G186), and F260 (I254), further strengthening the importance of this region for function as RBD1 (Figure 3B, purple).

#### A unique receptor switch by LASV occurs after endocytosis

Initially, it was suggested that LASV cell entry occurred via clathrin-mediated endocytosis [144], but subsequent studies described the usage of a clathrin- and dynamin-independent pathway [6,76,145,146]. Genome-wide RNA interference silencing screens identified sodium hydrogen exchangers (NHEs), which have previously been implicated in macropinocytosis, as host factors involved in the multiplication of LCMV [147,148]. In a successive study, NHE was validated to be the entry factor and was thus indicative that LASV enters the host cell through macropinocytosis [146,149]. This type of endocytosis involves the nonspecific uptake of extracellular molecules via large vacuoles [150]. Intriguingly, macropinocytosis is the entry pathway for over 20 viruses, including HIV, herpes simplex virus 1, and EBOV [151–156].

Upon macropinocytosis, the virion is internalized and delivered to the late endosome where fusion ultimately takes place. Studies have shown that LASV-mediated fusion occurs at an unusually acidic pH of below 5.0 with optimal fusion suggested to occur at a pH of ∼4.0 [157–159]. While extremely low pH is likely sufficient enough to trigger fusion, successful and efficient LASV entry requires GP1 to undergo a unique receptor switch from αDG to LAMP1 [78]. Like αDG, LAMP1 must be glycosylated for this engagement to occur as previous studies showed that an N-linked glycan in this receptor was a critical determinant in LASV binding [78,160]. Binding to LAMP1, which is localized to the inner membrane of the late endosome/lysosome, shifts the pH of optimal fusion to be less acidic [159,161]. Engagement with LAMP1 thus increases the efficiency of LASV fusion, but the exact role remains to be clarified. No other OW arenavirus has been shown to interact with LAMP1, regardless of full conservation of the LAMP1-binding site in GP1. This receptor switch is thus unique to LASV [160]. Thus far, the only other virus shown to utilize late endosome receptor-assisted fusion is EBOV, but again the exact mechanism remains unclear.

It has been shown that in the low pH environment of the late endosomal compartment, GP1 undergoes irreversible conformational changes that decrease its affinity for αDG while increasing its affinity for the acidic LAMP1 receptor [78,161,162]. Structural and mutational analysis has revealed a highly conserved histidine triad (H92/93/230) in GP1 that forms the LAMP1-binding site and second receptor-binding domain (RBD2) (Figure 3C, yellow) [139,163–165]. Protonation in this triad, specifically H230, has been proposed to facilitate conformational changes that expose the LAMP1-binding site [164–166]. Curiously, GP1 glycosylation motif at N89 was reported to shield the histidine triad in the pre-fusion conformation before being redirected after endocytosis to allow for LAMP1 binding (Figure 3C, black) [51,71,167]. More specifically, H92 packs against N89 at physiological pH, but N89 is shifted ∼10 Å away at low pH [101]. This further supports the notion that glycosylation is involved in receptor recognition and binding by GP1. Moreover, at neutral pH, H93 makes a hydrogen bond with the main chain oxygen of N90 [71]. When the pH is reduced, H93 rotates such that it is no longer capable of making this hydrogen bond. It thus appears that modifications in the location of the glycosylation motif at N89 and hydrogen bonding result in the opening of the binding site so that the histidine triad may engage with LAMP1. Additionally, four other residues (L84N, K88E, L107F, and H170S) that neighbor the histidine triad were identified that decrease LAMP1-binding affinity when mutated and thus may be involved in the binding site as well [168]. It is interesting that another histidine may be important in LAMP1 binding that exists outside RBD2.

To summarize, GP1 serves as the R for arenaviruses. GP1 will first engage with the host cell receptor on the plasma membrane, primarily αDG, then be endocytosed via macropinocytosis. Fusion occurs upon the virion’s delivery to the late endosome. In LASV, this is a multistep process that involves a receptor switch from the primary receptor, αDG, to the intracellular receptor, LAMP1. We anticipate future experimentation will likely delve into why LASV is the only arenavirus to utilize assisted fusion and a receptor switch. Previous work has already suggested that engagement with LAMP1 increases fusion efficiency, but whether this has any implications in LASV morbidity remains unclear. Investigation into the underlying molecular mechanisms of immune evasion and the residues involved in this interaction may answer this question. Additionally, given GP1’s critical role in viral entry, deducing these mechanisms would have serious impacts on the development of therapeutic interventions.

### Membrane fusion is mediated by GP2

**Figure 4:**
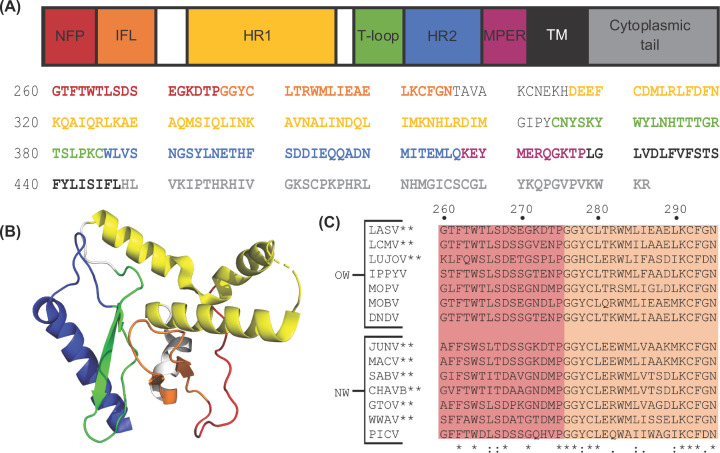
Sequence and structure of the LASV GP2 (**A**) The known domains of GP2 are color-coded: NFP (red); IFL (orange); HR1 (yellow); T-loop (green); HR2 (blue); MPER (purple); TM (black); cytoplasmic tail (gray). (**B**) Cartoon representation of the pre-fusion crystal structure of a GP2 monomer (PDB: 5VK2). (**C**) Sequence alignment of the NFP and IFL of pathogenic and/or OW arenaviruses with numbering according to the LASV sequence. Pathogenic arenaviruses are denoted via **. The prototypic Pichinde virus is also shown. Residues with full conservation are indicated by an asterisk (*), whereas those with strongly and weakly similar properties are shown via a semicolon (:) and period (.), respectively. Abbreviations: CHAVB, Chapare virus; DNDV, Dandenong virus; GTOV, Guanarito virus; IPPYV, Ippy virus; LUJOV, Lujo virus; MACV, Machupo virus; MOBV, Mobala virus; MOPV, Mopeia virus; PICV, Pichinde virus; SABV, Sabia virus; WWAV, Whitewater Arroyo virus.

GP2 is 231 amino acids in length and spans from G^260^ to R^491^ of the GPC (Figure 4A,B). Structurally, GP2 has the principle features of a class one fusion protein and is categorized as such due to its largely α-helical secondary structure and two heptad repeat (HR) regions that are preceded by a fusion peptide [59,81,169–171]. There are eight known domains of the GP2 including the NFP (G^260^–P^275^), IFL (G^276^–N^295^), heptad repeat 1 (HR1) (E^308^–S^358^), T-loop (C^364^–C^385^), heptad repeat 2 (HR2) (N^395^–I^411^), membrane proximal external region (MPER, T^412^–P^427^) transmembrane domain (TM, L^428^–L^447^), and C-terminal cytoplasmic tail (H^448^–R^491^) [9,157]. For this review article, we will be grouping these domains into three sections: fusion domain (NFP and IFL), coiled-coil (HR1, T-loop, and HR2), and C-terminus region (MPER, TM, and cytoplasmic tail).

#### Unique properties of the LASV fusion domain

LASV GP2 follows the generally accepted model for the function of class one viral fusion proteins where the fusion mechanism is mediated by its refolding to form a 6HB. For LASV, this occurs after GP1 dissociation where GP2 is uncovered so that the fusion peptide is now exposed. Class one viral fusion proteins all contain a hydrophobic sequence that is responsible for engaging the target membrane and thus bridging the host and viral membranes. Typically, this is either an NFP or IFL located within the fusion subunit of the surface glycoprotein. Most often, class one fusion proteins have been found to contain an NFP, except for EBOV which has an IFL [172]. However, in the case of LASV and the arenavirus family, both an NFP and IFL are present and create a fusion domain that initiates fusion by targeting the host cell membrane [157,173,174]. Thus far, the only other class one fusion protein with this characteristic are coronaviruses, potentially contributing to their remarkable infectivity [83–88].

The LASV fusion domain is moderately conserved among all OW and/or pathogenic arenaviruses (Figure 4C). Furthermore, the canonical fusion motif Gly-X-Phe located at the N-terminus of the fusion domain is also highly conserved among OW arenaviruses where X is either T or L [175]. This motif has been established in fusion peptides of other viruses, like HIV and influenza [176]. Single-point mutations to alanine for each hydrophobic amino acid in the fusion domain resulted in a significant reduction or abolishment of fusogenicity, validating the involvement of both the NFP and IFL in fusion [157]. Multiple residues were shown to impact fusogenicity through *in vitro* studies including G260A/R, F262A, W264A, G271A, which are all located in the NFP, and G277A, Y278A, L280A, W283A, L290A, and G294A located in the IFL. Four of these residues are well conserved across all arenaviruses and were indicated as critical for efficient fusion: W264, G277, Y278, and L280. Mutations in this region likely reduce the hydrophobicity of the fusion domain, which inhibits adequate insertion into the target membrane to facilitate fusion. This further strengthens the postulation that both the NFP and IFL are required for efficient fusion.

Nonetheless, the necessity for both fusion peptides in LASV and their exact mechanism of action remains poorly understood. The pre-fusion structure of these two proteins has been characterized as a random coil with the only discernible secondary structural element being a disulfide bond [71]. On the other hand, these proteins have been omitted from post-fusion structures of GP2 and thus remains unsolved [169,170]. Thus, future experiments will likely be centered around solving the post-fusion structure of the fusion domain to fill this knowledge gap. We anticipate that subsequent studies will investigate the residue-specific interactions that are critical to the function of the LASV fusion domain.

#### Coiled-coil structure further classifies LASV as a class one fusion protein

**Figure 5:**
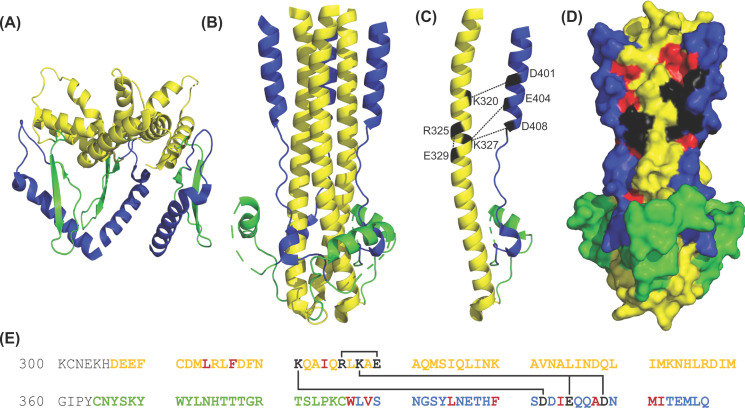
Representation of the LASV coiled-coil in different states (**A**) The pre-fusion state of the LASV HR1 (yellow) and HR2 (blue) trimers that exist independently but are connected by the T-loop (green). No helical core is present with the trimers oriented nearly perpendicular to the threefold axis (PDB: 5VK2). (**B**) In the post-fusion state, the HR1, HR2, and T-loop unravel and reorient to form the characteristic coiled-coil/6HB. A central trimer of HR1 is surrounded by three HR2. Each monomer is connected by a T-loop (PDB: 5OMI). (**C**) A monomer of the coiled-coil is presented with the residues that form salt bridges indicated in black. (**D**) A surface model of the coiled-coil (PDB: 5OMI). The hydrophobic interactions (red) between adjacent HR1 and HR2 monomers of the 6HB form a core that is further stabilized by salt bridges (black). (**E**) The sequence of the LASV HRs as connected by the T-loop. Residues that form salt bridges are shown in black and linked to their corresponding ionic partner, while residues involved in the hydrophobic core are shown in red.

Now that we have finished discussing the LASV fusion domain, we must highlight another major defining characteristic of class one fusion proteins—the formation of the 6HB. In both their pre- and post-fusion states, class one glycoproteins exist as trimers. In the pre-fusion state, the HR1 and HR2 trimers exist independently as α helices that are connected by the T-loop (Figure 5A). The T-loop itself does not appear to have a function aside from connecting the HR1 and HR2 [177]. Following the initiation of membrane fusion, a prehairpin intermediate structure is formed where the HR1 sits atop the HR2 in an antiparallel manner. HR1 and HR2 coordinate with each other to form the coiled-coil where a central HR1 trimer is surrounded by three HR2. The intermediate structure spontaneously collapses on itself where the three HR2 pack on to the outside of the central trimeric HR1 core to create the 6HB (Figure 5B). This formation of the 6HB is energetically favorable and the major driving force for fusion as it brings the target and virion membranes nearby such that they may fuse. Ultimately, the final post-fusion conformation of the trimer-of-hairpins is achieved, and a complete fusion pore is formed.

Typically, in class one fusion proteins the trimer is formed about a stabilizing central three-helical core with a threefold axis that remains largely unaffected between the pre- and post-fusion conformations [95]. Nonetheless, a pre-fusion LASV crystal structure was recently generated that demonstrated this core is nonexistent and GP2 is oriented nearly perpendicular to the threefold axis in the pre-fusion conformation (Figure 5A) [71]. To achieve the post-fusion conformation, every section of GP2 must thus unravel and reorient. The structure presented here aligned well with a previously determined structure of the LCMV GPC monomer. This reorientation is thus unique to arenaviruses [95]. Furthermore, alanine substitutions in JUNV at I333 (V341), L336 (L344), I347 (L355), and L350 (I358) in HR1 and R392 (S400) and W395 (I403) in HR2 resulted in reduced fusogenicity of the GP2 [178]. This infers that these buried residues are important in promoting fusion. In the pre-fusion conformation, these residues are positioned such that they have interhelical interactions that likely result in the stabilization of the coiled coil. In particular, R392 (S400) may impart specificity to the process of coiled-coil folding at the expense of protein stability [179].

In the post-fusion crystal structure, there are three HR1 helices in a coiled-coil orientation with three HR2 helices entwined in an antiparallel manner forming the 6HB [169,170]. The three HR1 helices were shown to be closely packed against each other by hydrophobic force in a parallel manner and create a central core [169]. The C-terminal HR2 adopts such an orientation that places its helix close to the N-terminal HR1 helices of the adjacent monomers. This positioning ultimately creates a hydrophobic core involving A407 and I411 of the HR2 and L313 and F316 of the adjacent HR1 (Figure 5C,D, red) [170]. The T-loop region and HR2 are packed against the hydrophobic grooves of the central coiled-coil. W386, V388, L394, and F399 of HR2 are deeply buried in these grooves of HR1 with many other long-chained, hydrophobic residues, such as I403 and M410, involved as well (Figure 5C,D, red). Granted, for stability purposes, only the HR1, T-loop, and HR2 domains of the LASV GP2 were included in this crystal structure [169] but aligned well with a similar reported LASV 6HB structure [170]. A separate study validated the necessity of L394 and I403 for GP2 function [9]. Additionally, using alanine scanning mutagenesis, I323 was identified as being critical for fusion and optimal GP2 function. This trend can likely be justified by the loss of hydrophobicity in this region, which does not allow for the efficient formation of the 6HB.

In addition to the hydrophobic interactions, several electrostatic interactions stabilize the 6HB. D347 and K352 of one HR1 domain bind to residues of opposite charges in the other two HR1 domains near the T-loop [169]. This interchain salt bridge was also found in the corresponding residues of LCMV and seemed to stabilize the end of the inner core [81]. Additional work on LCMV elucidated other stabilizing interactions, particularly multiple ionic interactions, which seem to also exist in LASV based on sequence homology and structural alignment. Two separate salt bridges exist between lysine side chains in the N-terminal helix and aspartate and/or glutamate side chains in the C-terminal helix. These are K326 (K320) with D414 (D408) and K333 (K327) with both D407 (D401) and E410 (E404) (Figure 5C–E, black). K331 (R325) and D335 (E329) form a salt bridge to connect two consecutive turns in the N-terminus helix (Figure 5C–E, black). These salt bridges are thus well conserved and aid in stabilization between GP2 monomers of the post-fusion conformation.

#### C-terminus region has multiple domains that are key to LASV fusogenicity

Our review of GP2 will conclude with a discussion of the C-terminus domains and their roles in fusogenicity. This includes the TM, cytoplasmic tail, and MPER, which is located between HR2 and TM from residues T^412^ to P^427^ (Figure 4A) [180]. In class one fusion proteins, namely EBOV and HIV, the MPER has been shown to have important functions in fusion and glycoprotein stabilization [181–186]. The MPER in HIV is the target of the broadest neutralizing antibodies and has implications in membrane fusion due to destabilizing effects on the lipid membrane [187–192]. Additionally, in EBOV, the MPER was shown to interact with its IFL and membrane lipids during fusion—potentially contributing to the driving force of 6HB formation to promote fusion [181,193]. Following this logic, the MPER in GP2 has been investigated for any impacts on LASV fusogenicity.

**Figure 6:**
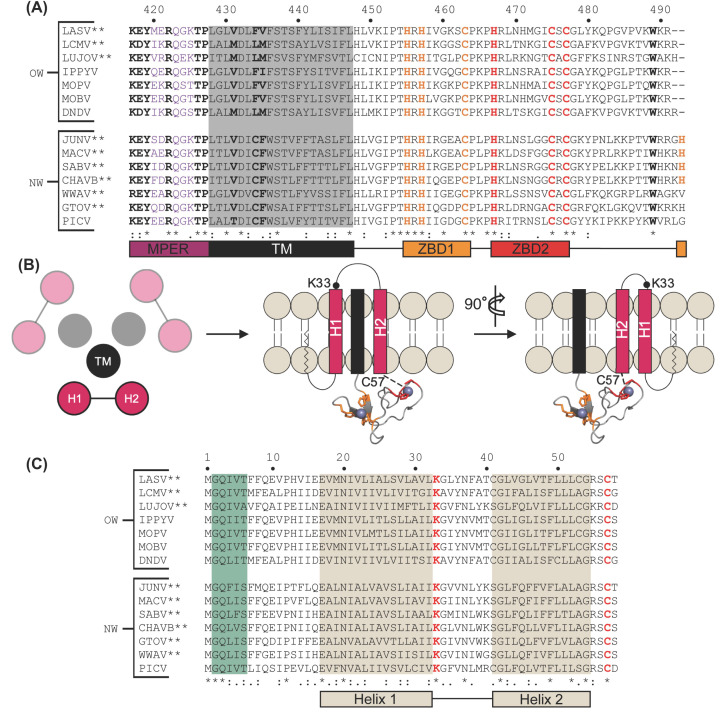
Characterization of the SSP and cytoplasmic tail of GP2 (**A**) Sequence alignment of the LASV GP2 C-terminus of pathogenic and/or OW arenaviruses. Residues in the MPER (purple), TM (shaded black), and cytoplasmic tail implicated in fusion are bolded. The ZBD1 (orange) and ZBD2 (red) are shown. (**B**) Illustration of the SSP (pink) and its association with the TM (black) of GP2 from the top and side view. Helix 1 (H1) creates an interface with the TM, whereas helix 2 (H2) contains C57, the fourth ligand in ZBD2 (red). ZBD1 (orange) is shown as well. Each ZBD coordinates with a zinc ion (purple) (**C**) Sequence alignment of the SSP with the myristoylation motif outlined in green. Both helical transmembrane regions are shaded in tan. K33 and C57 are shown in red. Pathogenic arenaviruses are denoted via **. The prototypic Pichinde virus (PICV) is also shown. Residues with full conservation are indicated by an asterisk (*), whereas those with strongly and weakly similar properties are shown via a semicolon (:) and period (.), respectively. Abbreviations: CHAVB, Chapare virus; DNDV, Dandenong virus; GTOV, Guanarito virus; IPPYV, Ippy virus; LUJOV, Lujo virus; MACV, Machupo virus; MOBV, Mobala virus; MOPV, Mopeia virus; PICV, Pichinde virus; SABV, Sabia virus; WWAV, Whitewater Arroyo virus; ZBD, zinc-binding domain.

In LASV, the formation of the 6HB brings the fusion domain near the TM region, including the MPER, which may contribute to the driving force of fusion. This was investigated using alanine scanning mutagenesis of all 16 residues in the MPER [180]. It was revealed that M414 and L415 in the C-terminus of HR2 and K417 and Y419 in the N-terminus of the MPER were critical for membrane fusion (Figure 6A). Even though mature GPC was present with these mutants, it was nonfunctional and incapable of facilitating fusion. Mutation to a more conserved residue, such as M414L, L415I, K417R, and Y419F, resulted in partial recovery of fusogenicity. The finding that K417 is critical for GP2 function comes as no surprise since it is highly conserved and the equivalent lysine in Guanarito virus (GTOV), an NW arenavirus with pathogenicity in humans, forms a salt bridge with a conserved aspartate and glutamate in the adjacent HR1 [194]. Moreover, E418, R422, T426, and P427 all exhibited reduced fusogenic activity when mutated to alanine. R422 was validated as a critical residue for GP2 function in a separate study, but it is unclear if this residue forms a salt bridge, and if so, with what partner [9]. Altogether, this is indicative that the MPER is critical to the stability of GP2 with implications in efficient LASV-mediated fusion.

Directly following the MPER lies the TM. Unfortunately, structural characterization of the TM has been challenging—largely due to its hydrophobic nature. Instead, critical residues in the TM have been identified through inhibitor screens. The TM has been shown to have an interaction with the first helix of the SSP, which forms an interface that is often the target of inhibitors and has been proposed to have a role in the pH-sensing mechanism [27–29,39,195]. The mechanism of action behind these inhibitors seems to be the stabilization of the pre-fusion GPC complex via the TM–SSP interface as revealed by inhibition studies [195–197]. The dietary supplement capsaicin [28] and the antifungal isavuconazole [27] were both revealed to target the same three residues in the TM: V431, F434, and V435. Capsaicin and isavuconazole targeted the adjacent region within the N-terminal transmembrane region of the SSP at A25 and S27, respectively. Curiously, the mutation F427I (V435) in the TM of the JUNV GP2 confers immunological resistance to the live-attenuated Candid#1 vaccine [198]. This mutation has an epistatic interaction with a lysine-to-serine mutated residue in the SSP (K33), a residue that is critical for the SSP’s role in pH-sensing [198,199]. These mutations are the sole basis for the vaccine’s attenuation and have thus raised concerns for reversion to the pathogenic phenotype. Regardless, more structural information is necessary to validate this hypothesis. Like the fusion domain, the pre-fusion and post-fusion state of the TM remains to be clarified. Elucidation of this structural information will shed some light on the interactions within the TM-SSP interface and their roles in the functionality of the GP2.

The retention of SSP is dependent not only on its interaction with the TM but the cytoplasmic tail as well. This was inferred as replacement of the TM and cytoplasmic tail with an unrelated sequence impacted SSP association, whereas substitutions elsewhere in the GPC did not [75]. Furthermore, the cytoplasmic tail of JUNV contains six cysteine and histidine residues that are essential for SSP association and form two different zinc-binding domain (ZBD) motifs [200–202]. The first ZBD (ZBD1) is formed by H447 (H455), H449 (H457), and C455 (C463), whereas the second ZBD (ZBD2) is created by H459 (H467), C467 (C475), and C469 (C477) (Figure 6A). These two ZBD motifs are completely conserved across all NW and OW arenaviruses (Figure 6B). Using isothermal titration calorimetry, ZBD1 and a nonconserved H485 were first identified to bind zinc with a dissociation constant (*K*_d_) in the nanomolar range [200,201]. Oddly, H485 is absent from all NW and OW arenaviruses that are not within the same group as JUNV, including LASV (Figure 6B). This lack of H485 raises the postulation of what might complete the tetrahedral structure. Is it water like ZBD2 before the SSP association? Could it be another residue within the SSP, such as C41 which is moderately conserved? Probably not considering mutational analysis revealed H485 was important in the coordination of the JUNV ZBD but was not necessary for the retention of SSP or membrane fusion activity. What about other conserved residues within GP2? Nonetheless, there is no obvious answer to these questions as no other histidine or cysteine residues are strictly conserved across all arenaviruses.

To follow-up, York and colleagues hypothesized that ZBD2 was formed with C57 of the SSP as the fourth ligand (Figure 6C) [201]. Before SSP association, a water molecule acts as a fourth ligand to complete the tetrahedral coordination of zinc. The necessity of these zinc-binding residues was further validated in a separate study on Pichinde virus, a nonpathogenic NW prototypic arenavirus [202]. In the same study, it was found that the fully conserved W503 (W488) was also indispensable for viral replication. Individual mutational studies on all seven of these residues (H447, H449, C455, H459, C467, C469, and W503) resulted in the complete abolishment of SSP association and rendered the GPC inactive [200–202]. It was thus speculated that these residues either facilitate the proper folding of the cytoplasmic tail and its association with the SSP or merely that zinc coordination is required for SSP retention. The rationale for the former will be discussed more in-depth later in this review article.

Altogether, this affirms the characterization of the LASV GP2 as a class one fusion protein as well as highlights some interesting features. Unlike other class one fusion glycoproteins, LASV GP2 contains a noncanonical NFP and IFL that must be characterized further to completely understand their necessity. The TM also requires further experimentation, including structural investigation and functional analysis. Interestingly, multiple inhibitors seem to target the same region (residues 434 to 437) in the TM of various OW and NW arenaviruses. This could potentially be a region of crucial hydrophobic interactions between the TM-SSP interface. Moreover, an investigation is also necessary to fully probe the novel ZBDs, specifically the fourth ligand of ZBD1 in LASV and other arenaviruses. Other future directions will likely include characterizing the exact role of the MPER, how the GP2 coordinates with the SSP, and any conformational changes that are undergone during the fusion process.

### Unusual retention of the SSP in the mature GPC

Although the structural changes undergone by the LASV GPC during membrane fusion follow the same framework as other class one fusion proteins, the pH-induced activation in the late endosome is distinct—in part due to the unusual retention of the SSP in the mature GPC and its involvement in pH sensing. Conventional signal peptides are 18–30-amino acid peptides and are responsible for directing proteins to the proper intra- and/or extracellular location [203–207]. After successful translocation, the signaling peptide is cleaved and degraded. They usually contain a positively charged N-terminus, central hydrophobic region, and polar C-terminus [208,209]. On the contrary, the LASV SSP is abnormally long at 58 amino acids and spans from M^1^ to T^58^ of the mature GPC (Figure 6C). It is proposed to traverse the membrane twice with two distinct hydrophobic domains: helix 1 (H1, E^17^–L^32^) and helix 2 (H2, C^41^–G^54^). These helices have an antiparallel orientation and are connected by an eight amino acid ectodomain [89–91,199,210–213]. The N- and C-termini extend from M^1^–E^16^ and R^55^–T^58^, respectively, and reside in the cytosol [91,199].

Co-immunoprecipitation experiments have also demonstrated that the LASV SSP was retained in the mature GPC—a feature that is unique to arenaviruses [73,107,199]. After SPase cleavage, the SSP remains noncovalently associated with the GPC complex via the TM and ZBD2 [90,91,196,200,201]. Confocal microscopy studies on JUNV have demonstrated that the SSP is required for translocation of the GP1 and GP2 to the Golgi apparatus [212]. When the SSP was replaced with a traditional signal peptide or altogether absent, the GPC precursor was retained within the ER and was not processed or functional [73–75,89,214,215]. Therefore, not only is the SSP unusually retained in the mature form of the GPC, but it is also an integral third subunit required for the proper maturation of GPC.

While the LASV SSP maintains the conventional role of a signal peptide for intracellular trafficking, it also has a role in the LASV pH-dependent fusion process. The association of the SSP with GP2 throughout membrane fusion has been shown to influence the pH-sensing mechanism and stabilize the intermediate conformation of GP2 [197]. More specifically, the highly conserved K33 within the SSP has been shown to be required for fusion activity of the GPC. When this residue was substituted for alanine, fusion was not observed, likely due to a complete loss of fusogenicity. Substitutions that reduced positive polarity, such as K33R, K33H, and K33Q, increased the threshold pH for fusion such that optimal pH was less acidic [196]. Intriguingly, K33 is located within the short ectodomain loop that links the two transmembrane helices of the SSP (Figure 6B,C) [75]. We postulate that this location positions K33 at a key location in the extracellular environment for sensing pH alterations and/or forming an intersubunit salt bridge with GP2.

The pH-sensing mechanism also seems to be influenced by ZBD2. As mentioned previously, four residues comprise the novel ZBD2 (C57, H459, C467, and C469)—one of which, C57, is located within the SSP [201]. This cysteine is invariable across all arenaviruses whereas residues flanking it are variable (Figure 6C) [90,201]. The variable residues are important for SPase cleavage and GPC maturation but are not responsible for SSP retention, whereas C57 has been shown to have roles in SSP retention [90,199]. Mutation at C57 resulted in the complete abolishment of SSP association with GP2 [200]. It could be speculated that zinc is required for SSP retention and the loss of the intersubunit ZBD hinders this coordination. However, other studies have shown that proper C57 placement is promoted by the N-terminal region of the SSP or transmembrane regions of the GP2 [212]. Thus, it is more so likely that ZBD2 coordinates with C57 to retain and position the ectodomain loop of the SSP appropriately [200–202].

Another unusual property is that the SSP is myristoylated at a conserved N-terminal myristoylation motif, specifically G^2^–T^6^ [73]. Myristoylation has been demonstrated to be an important modification for the viral lifecycle, membrane targeting, and conformational stability [216–225]. In the case of the LASV SSP, a point mutation to alanine at G2 resulted in a significant hindrance of membrane fusion activity while GPC assembly was unaffected [91,226,227]. Previous studies have also demonstrated that myristoylation does not appear to affect SSP retention, either. The mechanism of action behind this motif is unclear but has been suggested to function late in the fusion process to promote the merging of the membranes [227].

Overall, these studies have suggested the LASV SSP has multiple unique properties when compared with other class one fusion proteins and signal peptides. Based on the lack of GPC processing in the absence of SSP, it is evident that SSP retention is required for proper GPC maturation. Aside from this, however, there is an extensive knowledge gap surrounding the LASV SSP. Given its unusual retention in the mature form, it is difficult to draw direct comparisons to other class one fusion proteins. As a result, the structural and functional characteristics remain poorly understood. The identification of an intersubunit ZBD that is coordinated with C57 of the SSP and the ability of K33 to sense pH, both by coordinating with GP2, provides an interesting starting point for future experimentation. Based on the location of these residues, it seems a detailed understanding of the localized interactions between the SSP and GP2 will provide key insights into the unique function of SSP. Specific investigation into the role of myristoylation is also required. Together, these insights will improve our knowledge surrounding the unusual retention of the SSP, which may be helpful in the development of new therapeutics as multiple inhibitors seem to target the GP2–SSP interface.

## Conclusions

Despite LASV being the most prevalent arenavirus afflicting humans, there are limited treatment options due to a lack of structural and functional information. The GPC is the only protein located on the virion surface of the LASV particle and has a vital role in the virus’ lifecycle, which makes it an exciting target for drug development. In other class one fusion proteins, the incorporation of the viral particle is dependent on an interplay of interactions between the matrix protein and glycoprotein. However, before this can be fully understood in LASV, fundamental questions about the LASV GPC and the unique properties of its components must be answered. First, the molecular mechanism by which the glycosylated GP1 manages to evade antibody-mediated immune responses must be understood. The receptor switch by GP1 is also unique; thus, a deeper understanding of the residues involved in the receptor switch mechanism should be completed. The most extensive knowledge gap involves the LASV GP2 and SSP. Further experimentation must be completed on the fusion domain to dissect the requirement of two fusion proteins by LASV to facilitate fusion efficiently. Moreover, the structural features of the GP2 and SSP interaction, specifically the TM–SSP interface and novel ZBDs, must be addressed. Following this, the molecular mechanism by which the GP2 and SSP sense pH changes to promote membrane fusion should be elucidated. In terms of the SSP alone, the role of its myristoylation requires further investigation. Ultimately, these experiments will provide fundamental information for novel targets that will be instrumental in drug design against LASV.

## Abbreviations

αDG: α-dystroglycan
6HB: six-helix bundle
CFR: case fatality rate
CHAVB: Chapare virus
DNDV: Dandenong virus
EBOV: Ebola virus
GP1: glycoprotein 1
GP2: glycoprotein 2
GPC: glycoprotein complex
GTOV: Guanarito virus
HIV: human immunodeficiency virus
HR1: heptad repeat 1
HR2: heptad repeat 2
IFL: internal fusion loop
IPPYV: Ippy virus
JUNV: Junín virus
LAMP1: lysosomal-associated membrane protein 1
LASV: Lassa virus
LCMV: lymphocytic choriomeningitis virus
LUJOV: Lujo virus
MACV: Machupo virus
MOBV: Mobala virus
MOPV: Mopeia virus
MPER: membrane proximal external region
NFP: N-terminal fusion peptide
NHE: sodium hydrogen exchanger
NW: New-World arenavirus
OW: Old-World arenavirus
PICV: Pichinde virus
RBD1: receptor-binding domain 1
RBD2: receptor-binding domain 2
SABV: Sabia virus
SARS-CoV-2: severe acute respiratory syndrome coronavirus 2
SPase: cellular signal peptidase
SSP: stable signal peptide
TAM: Tyro3/Axl/Mer
TM: transmembrane domain
WWAV: Whitewater Arroyo virus
ZBD: zinc-binding domain
